# Efficacy of Texture and Color Enhancement Imaging in visualizing gastric mucosal atrophy and gastric neoplasms

**DOI:** 10.1038/s41598-021-86296-x

**Published:** 2021-03-25

**Authors:** Tsubasa Ishikawa, Tomoaki Matsumura, Kenichiro Okimoto, Ariki Nagashima, Wataru Shiratori, Tatsuya Kaneko, Hirotaka Oura, Mamoru Tokunaga, Naoki Akizue, Yuki Ohta, Keiko Saito, Makoto Arai, Jun Kato, Naoya Kato

**Affiliations:** 1 Department of Gastroenterology, Graduate School of Medicine, Chiba University, Inohana 1-8-1, Chiba-City, 260-8670 Japan; 2 Department of Medical Oncology, Chiba University, Chiba, Japan

**Keywords:** Gastroenterology, Medical research

## Abstract

In 2020, Olympus Medical Systems Corporation introduced the Texture and Color Enhancement Imaging (TXI) as a new image-enhanced endoscopy. This study aimed to evaluate the visibility of neoplasms and mucosal atrophy in the upper gastrointestinal tract through TXI. We evaluated 72 and 60 images of 12 gastric neoplasms and 20 gastric atrophic/nonatrophic mucosa, respectively. The visibility of gastric mucosal atrophy and gastric neoplasm was assessed by six endoscopists using a previously reported visibility scale (1 = poor to 4 = excellent). Color differences between gastric mucosal atrophy and nonatrophic mucosa and between gastric neoplasm and adjacent areas were assessed using the International Commission on Illumination L*a*b* color space system. The visibility of mucosal atrophy and gastric neoplasm was significantly improved in TXI mode 1 compared with that in white-light imaging (WLI) (visibility score: 3.8 ± 0.5 vs. 2.8 ± 0.9, *p* < 0.01 for mucosal atrophy; visibility score: 2.8 ± 1.0 vs. 2.0 ± 0.9, *p* < 0.01 for gastric neoplasm). Regarding gastric atrophic and nonatrophic mucosae, TXI mode 1 had a significantly greater color difference than WLI (color differences: 14.2 ± 8.0 vs. 8.7 ± 4.2, respectively, *p* < 0.01). TXI may be a useful observation modality in the endoscopic screening of the upper gastrointestinal tract.

## Introduction

Although the mortality rate of gastric cancer is decreasing worldwide, it still exceeds 800,000^1^. Despite the decreasing trend because of the widespread use of eradication therapy for *Helicobacter pylori* (*H. pylori*), gastric cancer remains the third leading cause of death worldwide^2,3^. In Japan, the incidence of gastric cancer ranks second among men and fourth among women^4,5^. Early detection of gastric cancer leads to early treatment, commonly endoscopic treatment. Its early detection promotes reduction in tumor invasion and prevention of cancer progression. The diagnosis of atrophic gastritis, which is a risk factor for gastric cancer, via upper gastrointestinal endoscopy is also vital. In upper gastrointestinal endoscopy, image-enhanced endoscopy (IEE) contributes to the detection of early gastric cancer and the diagnosis of atrophic gastritis; the most common IEEs are narrow-band imaging (NBI), blue-laser imaging (BLI), and linked color imaging (LCI)^6–13^. In particular, NBI facilitates the diagnosis and identification of early-stage gastric cancer by illuminating the mucosal surface structures at two narrow wavelengths (blue light at 390–445 nm or green light at 530–550 nm) that are easily absorbed by blood hemoglobin and highlighting the capillaries in the mucosal surface layer^14^. Similarly, BLI irradiates a narrow wavelength laser beam (450 ± 10 nm for white-light observation and 410 ± 10 nm for narrow-band observation) to the mucosa to highlight capillaries and fine patterns in the mucosa. In addition, the wavelength of the laser light is narrower than that of the narrow-band light of NBI, so the mucosa can be observed with higher specificity. Meanwhile, LCI increases the light output of the phosphor excited by enhancing the light output at 450 nm, making the screen brighter, and the image processing makes the color separation in the red region clearer. LCI also highlights the subtle color differences in the mucosa through imaging that further extends saturation and hue differences, thereby contributing to the diagnosis of inflammation and neoplasia^9–12^.

In 2020, Olympus Medical Systems Corporation (Tokyo, Japan) introduced a new IEE called Texture and Color Enhancement Imaging (TXI). In TXI, an image usually obtained by white-light irradiation is divided into texture image and base image, which are recombined after enhancing the texture and correcting the color tone and brightness. TXI optimizes three elements, namely, “structure,” “color tone,” and “brightness,” of the mucosal surface in ordinary white-light observation. Although TXI is currently expected to be useful, the conditions and lesions that can benefit from TXI in actual clinical practice are still poorly known. In addition, the efficacy of TXI remains unreported. TXI is expected to reveal subtle changes in mucosal irregularities, color tone, and vascular permeability, which are hardly detectable by white-light imaging (WLI), by emphasizing and correcting the color and contour of the mucosa. Therefore, this study aimed to assess the efficacy of TXI in visualizing gastric mucosal atrophy and gastric neoplasms.

## Materials and methods

### Instruments

This study employed the CV-1500 light source equipped with a TXI system, as well as the GIF-EZ1500 and GIF-H290Z endoscopes, (Olympus Medical Systems Corporation, Tokyo, Japan). TXI has two types: mode 1 and mode 2. In TXI mode 1, the overall image is slightly blue-white and the color tone changes are more coordinated than those in TXI mode 2, which has an orange color tone and provides an image similar to WLI. We also compared TXI mode 1 and mode 2 to contribute to appropriate mode selection. For the structure-enhanced mode, “A7” was selected for NBI and WLI, while “strong” was selected for TXI.

### Overview and features of TXI scans

**Figure 1:**
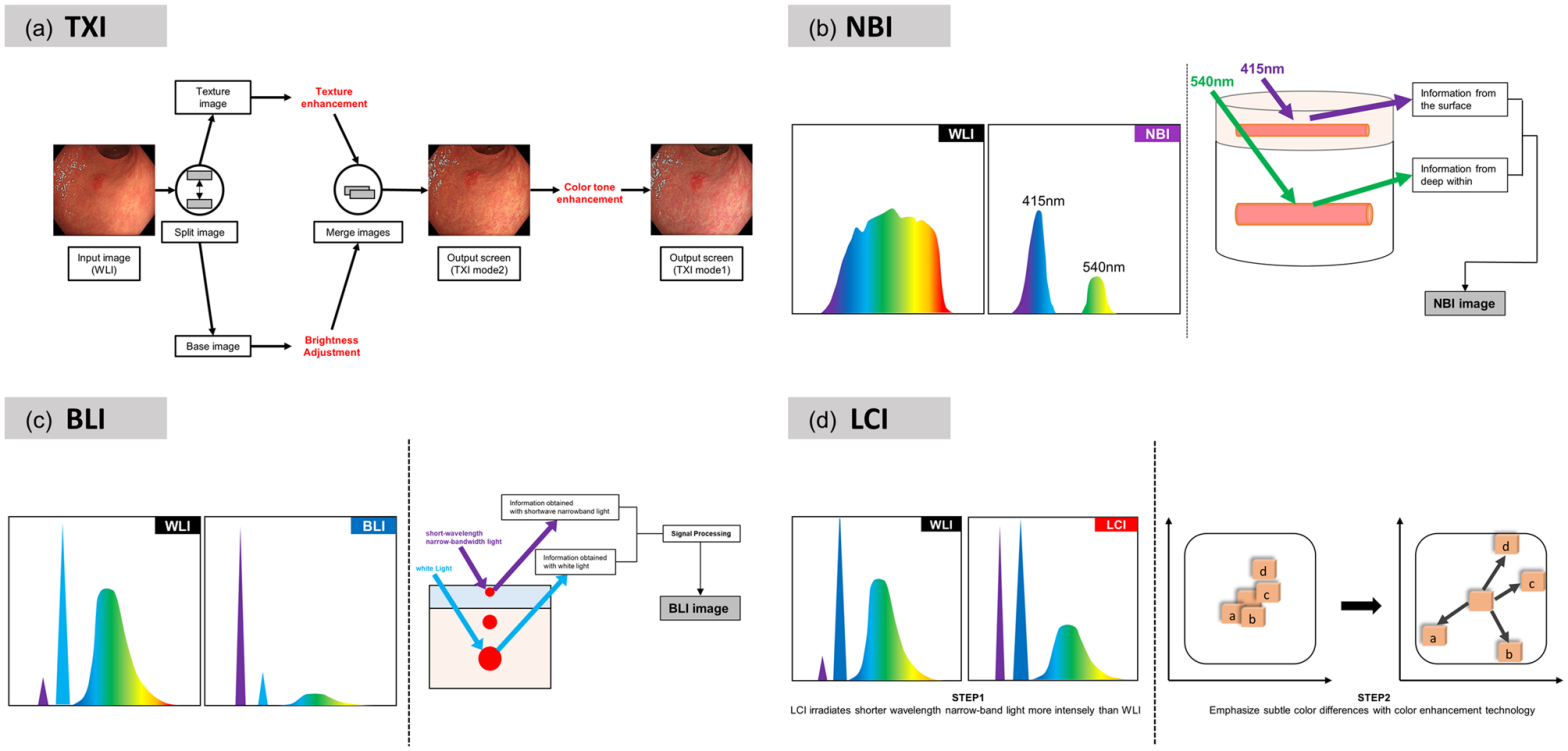
Overview and characteristics of images obtained using TXI and comparison with those of images obtained using other modalities: (**a**) TXI: In the first process of the TXI output, the image obtained from WLI is divided into a base image and a texture image. Then, texture enhancement is applied to the texture image and brightness adjustment is applied to the base image. These two images are combined to produce a TXI mode 2 image. Then, a TXI mode 1 image can be obtained by color enhancement of the TXI mode 2 image. On the other hand, NBI (**b**), BLI (**c**), and LCI (**d**) all obtain information by strongly irradiating a narrow band of light, which is thought to be more suitable for observing information on the mucosal surface and blood vessels. LCI is similar to TXI in that it uses color enhancement technology to enhance the visibility of lesions, while TXI differs from LCI in that it does not use more narrow-band light and adds texture enhancement.

The following is a description of the TXI scans: First, the image obtained from WLI is divided into a base image and a texture image. Then, texture enhancement is applied to the texture image and brightness adjustment is applied to the base image. These two images are combined to produce a TXI mode 2 image. Then, a TXI mode 1 image can be obtained by color enhancement of the TXI mode 2 image. Figure 1 shows the conceptual diagram of the TXI output.

### Study design and patients

This study enrolled consecutive patients who underwent esophagogastroduodenoscopy in July and August 2020 for gastric mucosal atrophy or gastric neoplasms, including early gastric cancer and adenomas, at Chiba University Hospital in Japan. A total of 23 patients were enrolled in the study after providing an informed consent; however, four patients were excluded because they could not obtain an adequate imaging set. Thus, we evaluated 19 patients with gastric mucosal atrophy and gastric neoplasms, including gastric adenomas or early-stage gastric cancer. All the patients were confirmed to have a current or history of *H. pylori* infection. In addition, 11 consecutive patients with 12 gastric neoplasms, who were examined during the same period, were evaluated. Although 15 gastric neoplasms could be imaged, three were excluded because appropriate images could not be successfully captured. Gastric mucosal atrophy was diagnosed endoscopically by two experts (T.I. and T.M.) using WLI, whereas gastric neoplasms were diagnosed by biopsy or endoscopic submucosal dissection. For both gastric mucosal atrophy and gastric neoplasms, images were taken continuously with WLI, TXI mode 1, and TXI mode 2 in the same composition. For gastric neoplasms specifically, images were also taken continuously after indigo carmine spraying, again with the same composition and imaging modes. The infection and eradication statuses of *Helicobacter pylori* were obtained from the patients’ medical records. Infection with *H. pylori* was regarded as positive if the record included at least one positive result from the urease breath test (Otsuka, Tokyo, Japan) or rapid urease test (Helicocheck; Otsuka) or indicated detection of *H. pylori* using serum IgG antibody (E-Plate “Eiken” *H. pylori* antibody; Eiken Chemical Co., Ltd., Tokyo, Japan)^15–17^. All the patients were confirmed to have a current or history of *H. pylori* infection. Endoscopic findings of gastric mucosal atrophy were evaluated in accordance with the Kimura–Takemoto classification^18^ The atrophic pattern was differentiated on the basis of the following features: C1, atrophic mucosa is only found in the antrum; C2, atrophic mucosa is found at the gastric angle or in the lower corpus; C3, atrophic mucosa is also found in the upper corpus; O1, atrophic mucosa surrounds the gastric cardia, but the folds of the great curvature are relatively maintained; O3, the entire gastric mucosa is atrophic, and the folds of the greater curvature as a whole disappeared; and O2, an intermediate condition exists between O1 and O3^13^.

This study was reviewed and approved by the Institutional Review Board of Chiba University School of Medicine and registered in the University Hospital Medical Information Network (UMIN000041436, approval date: July 1, 2020). All methods were performed in accordance with the relevant guidelines and regulations. Informed consent was obtained from each participant.

### Visibility score of gastric mucosal atrophy

**Figure 2:**
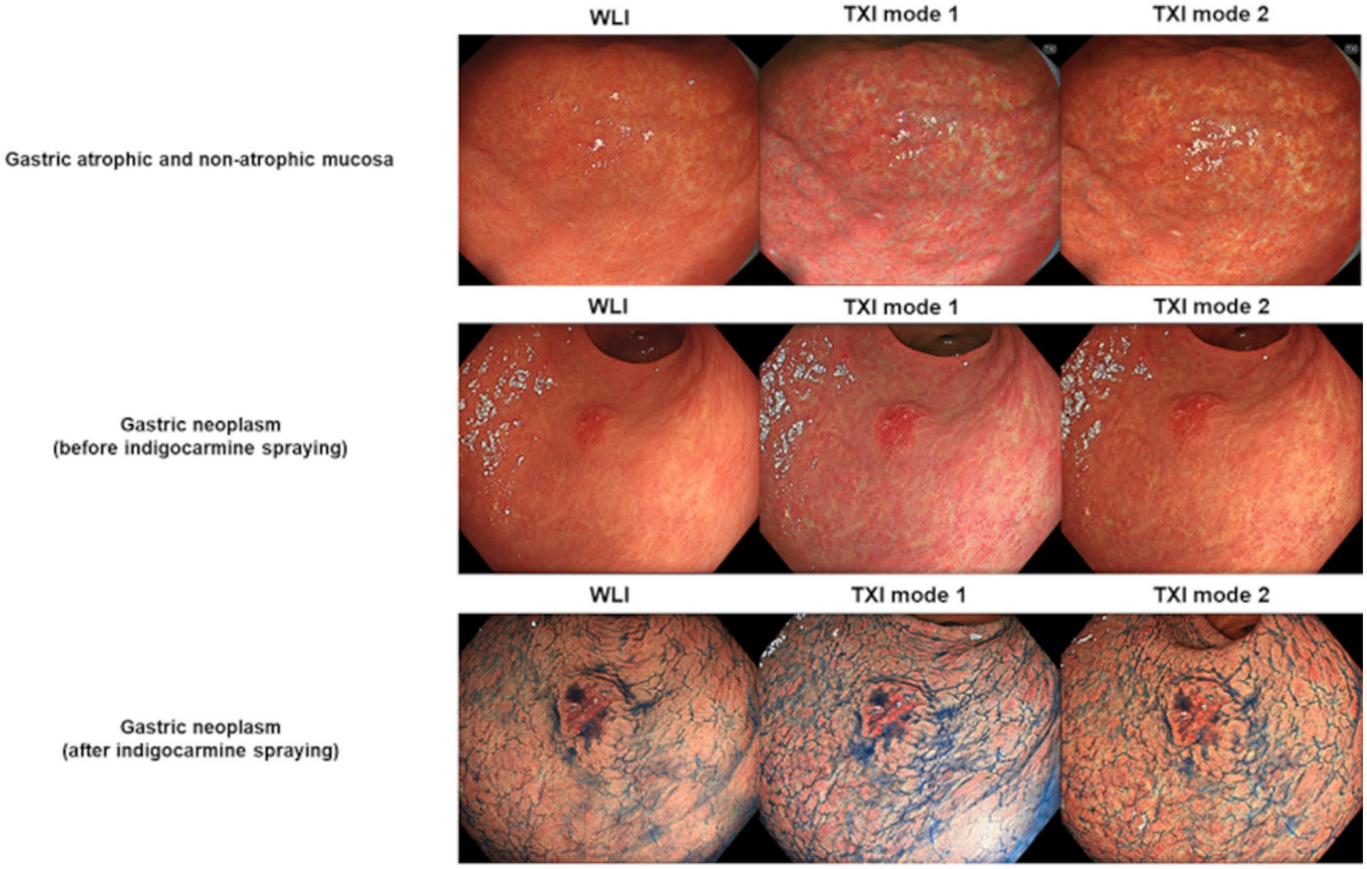
Examples of images of the gastric atrophic and nonatrophic mucosae and gastric neoplasm: The images were taken using WLI, TXI mode 1, and TXI mode 2 in the same composition in which the gastric atrophic and nonatrophic mucosae were observed simultaneously. Neoplasms were first imaged with WLI, TXI mode 1, and TXI mode 2 in the same composition. Indigo carmine was then sprayed on the neoplasms, which were subsequently imaged in the same mode.

**Figure 3:**
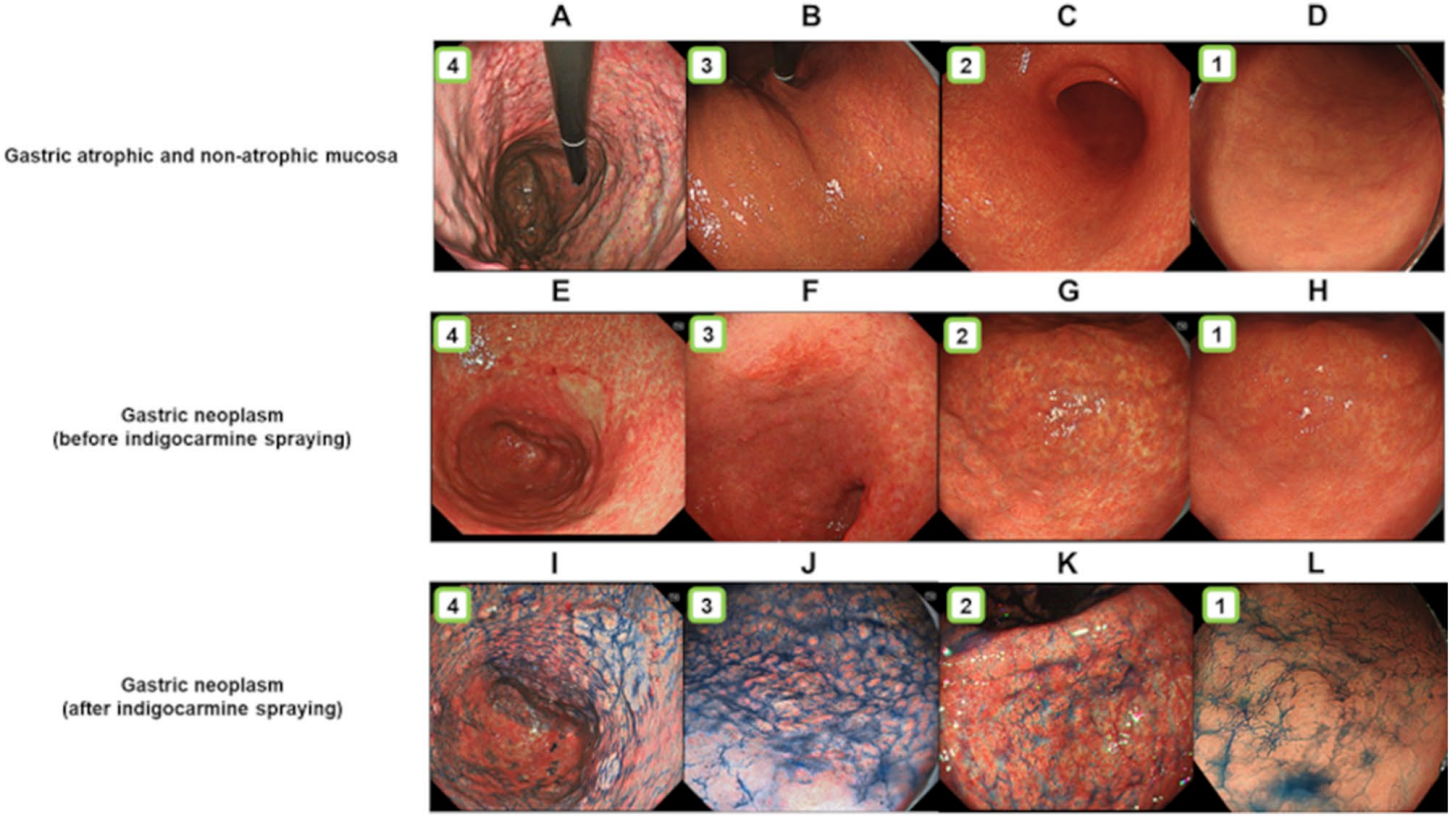
Examples of the visibility scores of gastric mucosal atrophy and the images obtained before and after spraying with indigo carmine: (**A**) an image with a visibility score of 4 taken in TXI mode 1; (**B**) an image with a visibility score of 3 taken in TXI mode 2; (**C**) an image with a visibility score of 2 taken in TXI mode 2; (**D**) an image with a visibility score of 1 taken using WLI; (**E**) an image with a visibility score of 4 taken in TXI mode 1; (**F**) an image with a visibility score of 3 taken in TXI mode 2; (**G**) an image with a visibility score of 2 taken in TXI mode 1; (**H**) an image with a visibility score of 1 taken using WLI; (**I**) an image with a visibility score of 4 taken using TXI mode 1; (**J**) an image with a visibility score of 3 taken in TXI mode 2; (**K**) an image with a visibility score of 2 taken in TXI mode 2; (**L**) an image with a visibility score of 1 taken using WLI.

In assessing the visibility score of the atrophic mucosa, first, images were taken in a composition that allowed single-image observation of the atrophic and nonatrophic mucosae. Images were acquired from the middle or distant view, with WLI, TXI mode 1, and TXI mode 2 in the same composition (Fig. 2). We assessed 60 images. Six endoscopists, including three experts and three trainees, interpreted the visibility of each image. Experts were defined as endoscopists who had at least 5 years of experience with IEE, and trainees were defined as residents with less than 1 year of experience as endoscopists. The endoscopists who diagnosed atrophic gastritis and those who subsequently evaluated the images were different. The reviewed images were presented electronically and without zooming. All images were fixed at the same size and reviewed on endoscopic monitors that were routinely used at the institution. Then, gastric mucosal atrophy was scored using a previously reported visibility scoring method^19,20^. The visibility scores for gastric mucosal atrophy were rated on a 4-point scale as follows: 4, excellent (can be easily determined); 3, good (can be detected with careful observation); 2, fair (can barely be detected); and 1, poor (cannot be detected with repeated observation). Figure 3 shows the representative images of each score.

### Visibility score of gastric neoplasms

In assessing the visibility score of gastric neoplasms, first, imaging was performed from the middle or distant view with the same composition for each lesion by WLI, TXI mode 1, and TXI mode 2. We assessed 36 images. Next, after spraying indigo carmine, we imaged the lesions in mid- or distant view using WLI, TXI mode 1, and TXI mode 2. Likewise, 36 images were obtained in this imaging (Fig. 2) and assessed by six endoscopists. Lesions were scored using a previously reported visibility score^11,19,20^. Visibility scores for gastric neoplasms were rated on a 4-point scale as follows: 4, excellent (easily detectable); 3, good (detectable with careful observation); 2, fair (almost undetectable upon close inspection); 1, poor (undetectable with repeated observation). Figure 3 depicts the representative images of each score.

### Color difference between gastric atrophic and nonatrophic mucosae

**Figure 4:**
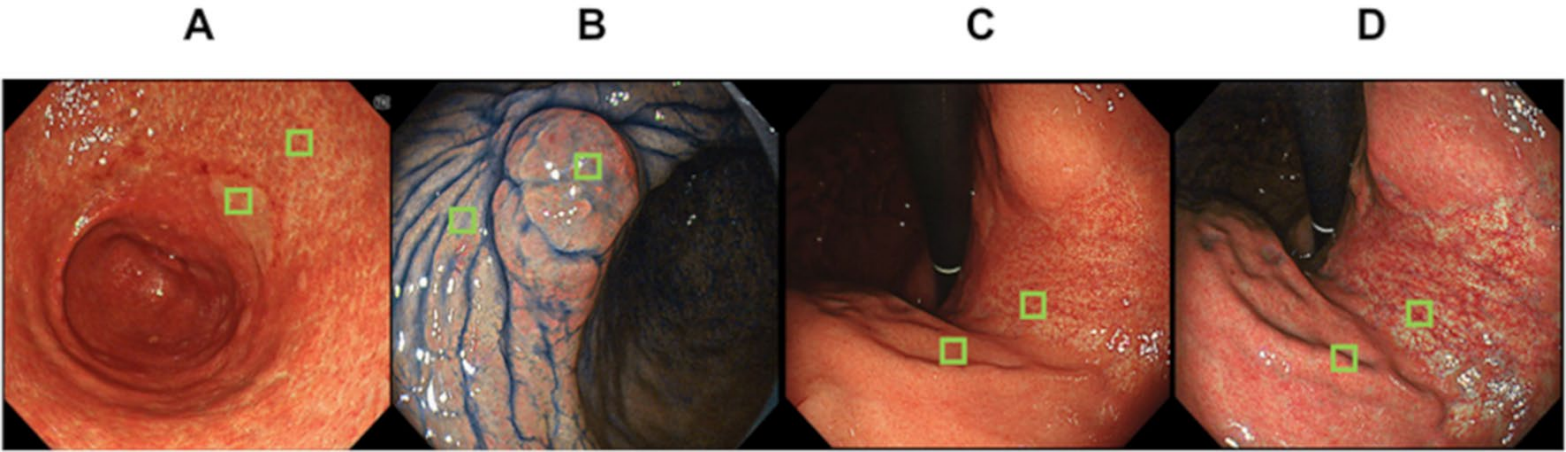
Examples of color sampling in the calculation of color difference: Color was sampled from atrophied and nonatrophied mucosae in atrophy determination. Similar colors were sampled from gastric neoplasms and non-neoplastic areas, and hue differences were calculated in the CIELAB color space system using Adobe Photoshop. (**A**) The area where color sampling is performed from the neoplasm and nearby non-neoplastic areas in the images taken in TXI mode 2. (**B**) The area where sampling is performed from the neoplasm and nearby non-neoplastic areas in the images taken in TXI mode 2 after indigo carmine spraying. (**C**), (**D**) Areas of color sampling in mucosal atrophic and nonatrophic areas taken using WLI and TXI mode 1, respectively.

Color differences (ΔE) were calculated using the International Commission on Illumination L*a*b* (CIELAB) color space system^21^. The CIELAB color space, which is a 3D model composed of a black-white axis (L*, brightness), a red-green axis (a*, the red-green component), and a yellow-blue axis (b*, the yellow-blue component), is designed to approximate human perception. The Euclidean distance between two points in the CIELAB color space is proportional to the difference in color perception. Here, we evaluated the perceived color difference in endoscopic images, with ΔE values calculated according to the CIELAB color space system^13,22–24^. We evaluated the color difference between gastric atrophic and nonatrophic mucosae by comparing the WLI scans with TXI mode 1 and TXI mode 2 images of the same composition taken consecutively (Fig. 2). We sampled 31 × 31 pixels of atrophied and nonatrophied mucosa color from images at 567 × 526 pixels and used the average value for our study. In addition, color was sampled from the same location in WLI, TXI mode 1, and TXI mode 2 (Fig. 4). The color values of the sampled sites were evaluated and scored according to the L* a* b* color values in the CIELAB color space system using Adobe Photoshop CC 2017 as previously described^16,20^. To calculate the color difference, we used the following formula^24–26^: ΔE* = [(ΔL*)^2^ + (Δa*)^2^ + (Δb*)^2^]^1/2^. Furthermore, we excluded pixels affected by halation.

### Color difference between gastric neoplasm and non-neoplastic areas

Similar to how the color difference between gastric atrophic and nonatrophic mucosae was evaluated, we compared gastric neoplasm and non-neoplasm images taken consecutively in the same composition with WLI, TXI mode 1, and TXI mode 2. The WLI, TXI mode 1, and TXI mode 2 images were also obtained after indigo carmine spraying (Fig. 2).

### Statistical analysis

Clinical data were expressed as percentages, averages, and ranges. We calculated the mean and standard deviation (SD) of the visibility scores and color differences (ΔE). The mean visibility scores rated by all endoscopists (experts and trainees) were also analyzed. Scores between modalities were compared using the Wilcoxon signed-rank test. Moreover, *p* < 0.05 indicated statistical significance. All statistical data were analyzed using the SPSS v. 22.0 for Windows (IBM Japan, Tokyo, Japan).

## Results

### Patients

Nineteen patients with gastric mucosal atrophy and/or gastric neoplasm were evaluated (12 men and 7 women; mean age, 73.0 ± 9.0 years). On the basis of the Kimura–Takemoto classification, mucosal atrophy was classified as follows: C1 in four patients (5.3%), C3 in one (5.3%), O1 in seven (36.8%), O2 in five (26.3%), and O3 in five (26.3%). Seven gastric neoplasms (46.7%) were found in the middle stomach and eight (53.3%), in the lower stomach. Regarding their morphology, nine cases (60.0%) were 0-IIa; four (26.7%), 0-IIc; and two (13.3%), 0-IIa + IIc. The gastric neoplasms consisted of differentiated adenocarcinoma in 10 cases (76.9%) and adenoma in 3 cases (23.1%).

### Visibility score of gastric mucosal atrophy

Gastric atrophic mucosa was more visible in TXI mode 1 and in TXI mode 2 than in WLI (*p *< 0.01 on both comparisons). However, TXI mode 1 was significantly better than TXI mode 2 (*p* < 0.01). Table 1 summarizes the results of the visibility scores, including those for the experts and trainees. The visibility score demonstrated a similar trend in other comparisons.

**Table 1:** Mean visibility scores for gastric mucosal atrophy determination using WLI, TXI mode 1, and TXI mode 2.

Endoscopist	WLI	TXI mode 1	TXI mode 2	WLI versus TXI mode 1, *p* value	WLI versus TXI mode 2, *p* value	TXI mode 1 versus TXI mode 2, *p* value
All, mean ± SD	2.8 ± 0.9	3.8 ± 0.5	3.5 ± 0.7	< 0.01	< 0.01	< 0.01
Expert, mean ± SD	2.9 ± 0.8	3.8 ± 0.5	3.5 ± 0.7	< 0.01	< 0.01	< 0.01
Trainee, mean ± SD	2.7 ± 0.9	3.7 ± 0.6	3.4 ± 0.8	< 0.01	< 0.01	< 0.01

*WLI* white-light imaging, *TXI mode 1* Texture and Color Enhancement Imaging mode 1, *TXI mode 2* Texture and Color Enhancement Imaging mode 2, *SD* standard deviation, *NS* not significant.

*p* value: exact Wilcoxon signed-rank test.

### Visibility score of gastric neoplasm

Gastric neoplasm lesions were more visible in TXI mode 1 than in WLI (*p* < 0.01). Similarly, TXI mode 2 was better than WLI (*p* < 0.01). However, TXI mode 1 was significantly better than TXI mode 2 (*p* < 0.01). Table 2 lists the results of other comparisons after indigo carmine spraying and the evaluation results by experts and trainees.

**Table 2:** Mean visibility scores of the gastric neoplasms imaged using WLI, TXI mode 1, TXI mode 2 indigo-WLI, indigo-TXI mode 1, and indigo-TXI mode 2.

Endoscopist	WLI	TXI mode 1	TXI mode 2	Indigo-WLI	Indigo-TXI mode 1	Indigo-TXI mode 2	WLI versus TXI mode 1, p value	WLI versus TXI mode 2, p value	TXI mode 1 versus TXI mode 2, p value	Indigo-WLI versus indigo-TXI mode 1, p value	Indigo-WLI versus indigo-TXI mode 2, p value	Indigo-TXI mode 1 versus indigo-TXI mode 2, p value
All, mean ± SD	2.0 ± 0.9	2.8 ± 1.0	2.2 ± 0.9	2.4 ± 1.1	2.7 ± 1.1	2.6 ± 1.1	< 0.01	< 0.01	< 0.01	< 0.01	< 0.01	0.070
Expert, mean ± SD	2.0 ± 0.8	2.9 ± 0.8	2.3 ± 0.9	2.4 ± 1.1	2.9 ± 1.0	2.7 ± 1.1	< 0.01	< 0.01	< 0.01	< 0.01	< 0.01	0.050
Trainee, mean ± SD	2.0 ± 0.9	2.6 ± 1.1	2.3 ± 1.0	2.4 ± 1.1	2.6 ± 1.1	2.5 ± 1.1	< 0.01	0.022	< 0.01	0.050	0.166	0.627

*WLI* white-light imaging, *TXI mode 1* Texture and Color Enhancement Imaging mode 1, *TXI mode 2* Texture and Color Enhancement Imaging mode 2, *indigo-WLI* WLI after indigo carmine spraying, *indigo-TXI mode 1* TXI mode 1 after indigo carmine spraying, *indigo-TXI mode 2* TXI mode 2 after indigo carmine spraying, *SD* standard deviation, *NS* not significant.

*p* value: exact Wilcoxon signed-rank test.

### Color difference between gastric atrophic and nonatrophic mucosae

The color difference (ΔE) between gastric atrophic and nonatrophic mucosae was significantly higher for TXI mode 1 than for WLI (8.732 ± 4.235 vs. 14.235 ± 8.012, *p* < 0.01). TXI mode 1 also had a significantly higher color difference than TXI mode 2 (14.235 ± 8.012 vs. 9.95 ± 4.167, *p* = 0.017). Meanwhile, the color difference between WLI and TXI mode 2 was not significant (8.732 ± 4.235 vs. 9.95 ± 4.167, *p* = 0.261). These results are summarized in Table 3.

**Table 3:** Objective evaluations using color differences (ΔE*; mean ± SD**).**

	L* a* b* values	WLI	TXI mode 1	TXI mode 2	*p* value	*p* value	*p* value
					WLI versus TXI mode 1	WLI versus TXI mode 2	TXI mode 1 versus TXI mode 2
Gastric neoplasm versus non-neoplasm	ΔE*	8.0 ± 4.2	18.7 ± 16.0	10.2 ± 8.4	< 0.01*	0.831	0.042
Gastric neoplasm versus non-neoplasm after indigo carmine spraying	ΔE*	11.2 ± 8.0	28.1 ± 12.7	16.8 ± 11.2	< 0.01	0.151	< 0.01
Atrophied mucosa versus nonatrophied mucosa	ΔE*	8.7 ± 4.2	14.2 ± 8.0	10.0 ± 4.2	0.009	0.261	0.017

*WLI* white-light imaging, *TXI mode 1* Texture and Color Enhancement Imaging mode 1, *TXI mode 2* Texture and Color Enhancement Imaging mode 2, *indigo-WLI* WLI after indigo carmine spraying, *indigo-TXI mode 1* TXI mode 1 after indigo carmine spraying, *indigo-TXI mode 2* TXI mode 2 after indigo carmine spraying, *SD* standard deviation, *NS* not significant.

*p* value: exact Wilcoxon signed-rank test.

### Color difference between gastric neoplasm and non-neoplastic areas

The color difference (ΔE) between neoplastic and non-neoplastic areas was significantly higher for TXI mode 1 than for WLI [8.000 ± 4.263 vs. 18.728 ± 16.046, *p* < 0.01]. TXI mode 1 also showed a significantly higher color difference than TXI mode 2 [18.728 ± 16.046 vs. 10.246 ± 8. 379, *p* = 0.042]. Meanwhile, color difference between WLI and TXI mode 2 was not significant (8.000 ± 4.263 vs. 10.246 ± 8.397, *p* = 0.831). Table 3 lists the color differences, including the results after indigo carmine spraying. Furthermore, the color difference between gastric neoplasms and non-neoplastic areas before and after indigo carmine spraying was higher in TXI mode 1 than in other imaging modes, and WLI and TXI mode 2 had no significant difference. The color difference between the gastric neoplasms and non-neoplastic areas after indigo carmine spraying was highest in TXI mode 1.

## Discussion

The results of this study showed that screening endoscopy using TXI mode 1 may provide more visible gastric neoplasms, including early gastric cancer and gastric adenomas, and gastric mucosal atrophy than screening endoscopy using WLI. TXI mode 1 also seemed to be a better modality for visualizing lesions and gastric mucosal atrophy than the newly introduced TXI mode 2. To our knowledge, this study is the first to compare color assessment and visibility of gastric neoplasms and gastric mucosal atrophy using the CIELAB color space system and visibility score in TXI.

This study also examined the visibility and color differences of gastric neoplasms and gastric mucosal atrophy individually according to the assessments made by six endoscopists. Typically, endoscopists find lesions by using the unevenness of the mucous membranes and changes in color shades when examining the inside of the stomach. Hence, gastrointestinal endoscopy requires techniques that can detect slight differences in color shades. The same can be said for atrophic mucosa.

WLI is known to detect lesions that show clear differences in color shading or mucosal surface irregularities, but has difficultly recognizing lesions with little mucosal unevenness or only a slight change in color tone. To recognize such lesions, we need to detect the color difference between gastric neoplasm and non-neoplastic areas. TXI is a new technology that is expected to improve the visibility of these differences by emphasizing the color tone and structure, such as the outline, of the target lesion. The current study evaluated the objective color tone by using the CIELAB color space and visibility score for each imaging mode to assess the visibility of gastric mucosal atrophy and gastric neoplasm. Results showed that TXI mode 1 had a better contrast than WLI for the color difference between gastric neoplasm and non-neoplastic areas. Likewise, the contrast for the visibility of gastric mucosal atrophy was higher in TXI than in WLI. As mentioned, TXI has two modes, namely, mode 1 and mode 2, and TXI mode 1 showed a better contrast than TXI mode 2 in the visibility of gastric neoplasm and gastric mucosal atrophy. Even after the spraying of indigo carmine, which we frequently use for visualizing slight changes in gastric mucosa, TXI mode 1 also showed a better contrast than WLI or TXI mode 2 in the color difference. Moreover, the visibility score of TXI mode 1 was significantly higher than that of WLI and TXI mode 2. Therefore, TXI mode 1 is a better imaging method than WLI and TXI mode 2 for visualizing early-stage gastric cancer, adenomas, and gastric mucosal atrophy. The visibility scores of experts and trainees revealed basically similar trends. However, after indigo carmine spraying, the visibility ability of TXI mode 1 and TXI mode 2 was significantly increased compared with WLI when judged by experts. When judged by trainees, TXI mode 2 did not show a significant increase in visibility compared with WLI, possibly because of their lack of experience in observing indigo carmine sprays. TXI mode 1 is an image mode with a strong blue-white impression, and the blue color of indigo carmine and other substances are emphasized. Thus, TXI mode 1 may aid in detecting lesions when combined with indigo carmine spraying. However, TXI mode 1 requires some familiarity with the color tone of the images compared with WLI, which is more familiar among endoscopists. Meanwhile, TXI mode 2 is an orange-tinged image mode that is relatively close to WLI. The selection of imaging mode for screening upper gastrointestinal endoscopy depends on the decision of each endoscopist.

This study, however, has several limitations. First, it was a small, single-center study. Second, the number of images evaluated was small. Third, in terms of visibility, the decision was made according to still images rather than video. Fourth, endoscopy experts were clearly expecting that imaging modes other than WLI would have better visibility because of the nature of these modes. Fifth, we examined visibility, not detectability. However, considering our results that the use of TXI improved the visibility and color difference of gastric neoplasms, we believe that TXI has the potential to increase the detectability of these neoplasms. For future research, a larger sample size is required, and the detection rate of gastric neoplasms, including early gastric cancer and gastric adenomas, will need to be studied in a multicenter, prospective, randomized, controlled trial. Sixth, we did not compare TXI with other modalities such as NBI, BLI, and LCI. In this study, we focused on the evaluation of gastric mucosal atrophy and detection of gastric neoplasms in screening. For these reasons, we did not collect data that could be directly compared with NBI, which is commonly used for scrutiny. BLI and LCI are similar observation methods, but because we used Olympus endoscopes in this study, we could not compare LCI and TXI. The seventh limitation is the identities of the images themselves. TXI scans are outputted by processing images obtained from WLI scans. The images used in this study for color difference and visibility scoring were taken using TXI and WLI in the same composition and object in succession. The WLI and TXI scans used in this study were not created from the exact same WLI scan, but were images taken in succession.

In conclusion, TXI mode 1 is recommended for the endoscopic screening of the upper gastrointestinal tract. In addition, TXI mode 1 observation after indigo carmine spraying is useful for lesion visibility because of the enhanced blue coloration.
